# The Use of Cook Resonance Metallic Ureteral Stent in Cases of Obstructive Uropathy from Persistent Neoureteral Stenosis, Following Kidney Transplantation

**DOI:** 10.1089/cren.2017.0005

**Published:** 2017-03-01

**Authors:** Victoria Stainer, Rachel Jones, Sanjay Agawal, Iqbal S. Shergill

**Affiliations:** ^1^ The Alan de Bolla Urology Unit, Wrexham Maelor Hospital, Betsi Cadwaladr University Health Board, Wrexham, United Kingdom.; ^2^ Department of Radiology, Wrexham Maelor Hospital, Betsi Cadwaladr University Health Board, Wrexham, United Kingdom.; ^3^ The North Wales and North West Urological Research Centre, Wrexham Maelor Hospital, Betsi Cadwaladr University Health Board, Wrexham, United Kingdom.

**Keywords:** metallic ureteral stent, obstructive uropathy, transplant

## Abstract

***Introduction:*** Following kidney transplantation, persistent cases of obstructive uropathy from neoureteral stenosis, at the reimplantation site, may require management with permanent, long-term Double-J stenting, following failed open surgical and minimally invasive procedures. We report our experience of the use of Cook Resonance^®^ metallic ureteral stent to manage such cases endourologically.

***Materials and Methods:*** Medium-term follow-up of two cases requiring long-term ureteral stenting. Medical records, operative details, and radiologic data were reviewed. Primary outcome was relief of obstructive uropathy, and secondary outcomes included clinicoradiologic complications and cost–effectiveness of the metallic stents compared with standard Double-J stents.

***Results:*** Case 1 was a 45-year-old lady with obstructive uropathy after kidney transplantation. To date, she has had four metallic stents, and on review of operative details and radiologic data, there was seen to be a 60% reduction in operation length from the first to fourth stent exchange. Radiation dose exposure saw an 80% reduction from 2852 to 556 CGy·cm^2^. Following 3 years of follow-up, relief of obstructive uropathy has been maintained, with no radiologic or clinical evidence of complications. Case 2 was a 44-year-old lady with obstructive nephropathy requiring long-term stenting after kidney transplantation. Two stent exchanges have been performed to date with a 38% reduction in operation length from 50 minutes to just 31 minutes. Radiation dose exposure saw a 41% reduction. No clinicoradiologic complications or stent-related symptoms have occurred.

***Discussion:*** In our experience, use of metallic stents in transplanted kidneys is safe and feasible, with both patients having effective and sustained relief of obstructive uropathy. This stent appears to be well tolerated and is associated with minimal clinicoradiologic complications. Metallic stent replacement is also cost-effective due to the fact that it only requires annual rather than 6-monthly stent changes.

## Introduction

Following kidney transplantation for the treatment of end-stage kidney disease, one of the most common urologic complications is obstructive uropathy from neoureteral stenosis at the reimplantation site. The incidence of obstructive uropathy has been documented and is ∼2% to 10%. The consequences of obstruction can be extremely severe with there being a risk to the transplanted kidney if obstruction is not identified and treated effectively. Other results of obstruction include renal failure, infection leading to abscess formation or sepsis, urinary leak, or fistula formation.^1^

Once an obstruction has been identified, it usually requires a multidisciplinary approach involving surgeons and interventional radiologists and nephrologists. Multiple methods of treating such obstruction have been reported in the literature, including the use of long-term nephrostomy, long-term Double-J stents, cold knife incision of stricture, or open surgical techniques.^2^ Currently, the Double-J stent technique is most commonly used but has to be exchanged on a 6-monthly basis usually requiring regular general anesthesia. In addition, there is increased risk of infection, encrustation, and stone formation, as well as stent migration. The cold knife incision technique can be effective on short segment strictures. Nephrostomy provides good relief to urinary obstruction in an acute setting, but long term is not a definitive treatment as it requires regular changes and can be a source of infection. Open surgery can be used for definitive management but has a high risk of morbidity and mortality rate and may not be suitable for all patients.

Recently, we managed such a case with Cook Resonance^®^ metallic stent and now report our experience of this stent in two cases, with medium-term follow-up. We also included a cost analysis comparing the traditional Double-J stents *vs* the metallic stents in the management of long-term ureteral stenting, in such patients.

## Materials and Methods

We present medium-term follow-up case reports of the use of the Cook Resonance metallic ureteral stent in two patients requiring long-term ureteral stenting for obstructive uropathy after kidney transplantation. Medical records were reviewed, including operative details, radiologic data, and follow-up consultations, as well as length of radiation screening and exposure. We used literature searches to evaluate the cost–effectiveness of the metallic stents compared with the traditional Double-J stents.

## Presentation of Case

### Case 1

A 45-year-old lady underwent an uneventful live related donor renal transplantation, however, postoperatively she developed obstructive uropathy with marked hydronephrosis. Due to persistent obstruction, despite open surgery and following multiple distal stent migrations and expulsions, necessitating frequent nephrostomy insertion, she underwent an effective retrograde insertion of a 12 cm 6F Cook Resonance metallic ureteral stent, under general anesthesia.

There was difficult retrograde access of the transplanted ureter and initial stent insertion had a radiation exposure of 2854 CGy·cm^2^. Various techniques were attempted to pass the guidewire and insert the stent. These included the use of a rigid cystoscope, a semirigid ureteroscope, and a flexible cystoscope. We found the best way was to use the flexible cystoscope. Subsequent stent exchanges were easier and quicker to perform with the techniques being improved upon each time. We saw an 80% in the fourth stent exchange to 556 CGy·cm^2^. Total operating time for the first insertion was 140 minutes, which has progressively improved over the subsequent three further stent exchanges, with the most recent taking just 55 minutes (60% reduction).

Following 3 years of follow-up, relief of obstructive uropathy has been maintained. The patient tolerates the stent extremely well, with no radiologic or clinical evidence of complications. Annual stent exchange, on three separate occasions, has been uneventful, with significantly decreased operative and radiation exposure times (Table 1).

**Table 1.** Radiologic Exposure Case 1

	*First*	*Second*	*Third*	*Fourth*
Operation length (minutes)	140	70	70	55
Screening time (seconds)	313	301	304	80
Dose (CGy·cm^2^)	2854	2832	2967	556

### Case 2

**FIG. 1.**
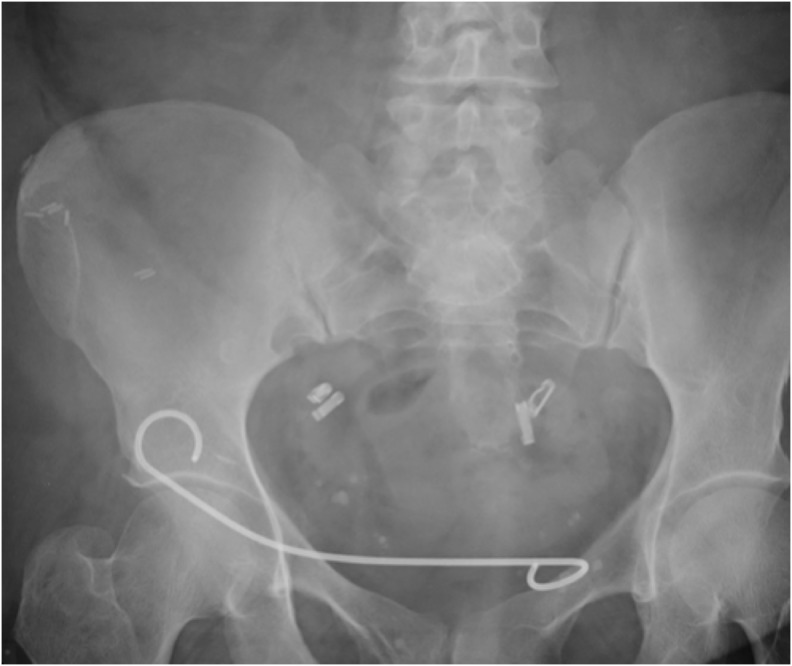
Cook Resonance^®^ metallic stent in transplanted ureter.

A 44-year-old lady underwent an uneventful cadaveric renal transplantation but postoperatively developed obstructive nephropathy requiring nephrostomy insertion, a laparotomy, and ureteral reimplantation. She experienced problems with a recurrent ureteral stricture requiring long-term management with Double-J stenting. Subsequently, she underwent stent exchange to the 12 cm 6F Cook Resonance metallic stent at our center (operating time 50 minutes and radiation exposure 1563 CGy·cm^2^. She has had a subsequent stent exchange with a significantly reduced operating time to 31 minutes (38% reduction), with a radiation exposure of 930 CGy·cm^2^ (41% reduction) (Fig. 1).

This dramatic reduction in operation time and radiation exposure is likely to plateau off with subsequent stent changes as after the initial insertion, the technique that works for that patient would hopefully be identified.

No clinicoradiologic complications or stent-related symptoms have occurred in follow-up. The current management plan is to exchange the metallic stent on a yearly basis as recommended by the manufacturer (Table 2).

**Table 2.** Radiologic Exposure Case 2

	*First*	*Second*
Operation length (minutes)	50	31
Screening time (seconds)	149	81
Dose (CGy·cm^2^)	1563	930

In our hospital when factoring the cost of different stents, guidewires, and the cost of an average theatre slot, the metallic stent came out £100 more cost-effective per year.

## Discussion

From our early experience, the use of metallic stents in transplanted kidneys is safe and feasible, with both patients having effective relief of obstructive uropathy. The resonance stent was initially designed for patients with malignant obstruction due to the fact that studies have shown it to be more resistant to compression than Double-J stents.^3^ In addition, the Cook Resonance stent has a unique coil design, allowing urine to drain even in cases of extreme compression. The resonance stent has occluded ends, and therefore, when exchanging the stent, a wire cannot be passed through the original stent. Passing a wire next to the stent is possible, but we found that this would always fall out when removing the initial stent.

This stent appears to be well tolerated and is associated with minimal clinicoradiologic complications. Other literature supports this with the Memokath ureteral stent being used in 73 patients with minimal rates of infection, stent dislodgement, and encrustation.^4^

Metallic stents are more expensive than the traditional Double-J stent, however, when you factor in that they only need annual exchange in our department we saw a £100 saving per year. Other studies have shown a better cost reduction with metallic stents, Taylor and colleagues showed a 48% to 75% reduction in costs per year with metallic stents.^5^ However, they were obtaining these values based on having three to six exchanges per year for the Double-J stent, which is different from our current practice. In 2009, a retrospective analysis of 13 patients showed that the Resonance stent was safe to use in patients for up to 12 months. This study found a 43% cost reduction.^6^
